# Phage phiKZ—The First of Giants

**DOI:** 10.3390/v13020149

**Published:** 2021-01-20

**Authors:** Victor Krylov, Maria Bourkaltseva, Elena Pleteneva, Olga Shaburova, Sergey Krylov, Alexander Karaulov, Sergey Zhavoronok, Oxana Svitich, Vitaly Zverev

**Affiliations:** 1 I.I. Mechnikov Research Institute of Vaccines & Sera, 105064 Moscow, Russia; mariabour@mail.ru (M.B.); epletmech@yandex.ru (E.P.); oshabs@mail.ru (O.S.); murk505@yahoo.com (S.K.); svitichoa@yandex.ru (O.S.); vitalyzverev@outlook.com (V.Z.); 2 Department of Clinical Immunology and Allergy, I.M. Sechenov First Moscow State Medical University of the Ministry of Health of the Russian Federation, 119146 Moscow, Russia; drkaraulov@mail.ru; 3 Department of Infectious Diseases, Belarusian State Medical University, 220116 Minsk, Belarus; zhavoronok.s@mail.ru; 4 Faculty of Preventive Medicine, I.M. Sechenov First Moscow State Medical University of the Ministry of Health of the Russian Federation, 119146 Moscow, Russia

**Keywords:** giant phages, phage phiKZ, phage particles structure, inner body, *Pseudomonas*, pseudolysogeny, phage therapy

## Abstract

The paper covers the history of the discovery and description of phiKZ, the first known giant bacteriophage active on *Pseudomonas aeruginosa*. It also describes its unique features, especially the characteristic manner of DNA packing in the head around a cylinder-shaped structure (“inner body”), which probably governs an ordered and tight packaging of the phage genome. Important properties of phiKZ-like phages include a wide range of lytic activity and the blue opalescence of their negative colonies, and provide a background for the search and discovery of new *P. aeruginosa* giant phages. The importance of the phiKZ species and of other giant phage species in practical phage therapy is noted given their broad use in commercial phage preparations.

## 1. Introduction

“Giant Phage”—The Origin of the Name

Phage phiKZ was the first bacteriophage to be classified as giant; it is active on *Pseudomonas aeruginosa* [1].

This phage has surprised researchers due to both the size of the phage particle (head diameter: 120 nm, tail length: 180 nm), and the size of the genome (280 kb) [1,2], which significantly exceeds the corresponding values of T-even phages of *Escherichia coli* (T2, T4, and T6). The latter were, for a long time, considered to be the bacterial viruses with the largest phage particles and genome sizes [3,4].

Using viral particle size for classification is quite natural and common [5], and the term “giant”, which emphasizes their unusualness, reflects the hope for new discoveries [6,7]. However, exactly what size of a viral particle should be considered as satisfactory for the use of the term “giant”?

It has been proposed that a new bacterial virus be considered as “giant or jumbo” if its genome size exceeds 200 kbp [8]. This value only slightly exceeds the genome size of T-even phages of *E. coli*, and, in our opinion, does not seem convincing enough to be used as a taxonomic criterion. The use of the physical size (or volume) of phage particles and their morphology may be necessary and possibly more preferable. Further to the importance of the morphology of viral particles for taxonomy, the existence of giant phages, for which genome length is significantly reduced with regard the theoretical capacity of the head of their particle, should be noted. 

The study of phage phiKZ, the phylogenetically related phages of *Pseudomonas* of different species, and giant phages of other bacterial species confirmed the relevance of the expectation of new discoveries. The appearance of many publications describing the research of new giant phages of different bacterial species [9,10,11,12,13,14,15,16,17,18] has significantly expanded the overall knowledge on the structure, properties, prevalence, and evolution of these phages. Thus, the continuing interest in the search for new species of giant phages, as well as the emergence of more detailed studies on the already described giants, is understandable. There is a desire to identify and understand the general and particular factors in the evolution of these phages which determine the tendency towards gigantism.

In this review we would like to focus on the early but very important findings obtained over the more than 40-year history of phiKZ and phiKZ-like *P. aeruginosa* phage studies (see Table 1).

## 2. Isolation and Growth Features of phiKZ

The phiKZ bacteriophage was discovered during the search for new phages suitable for phage therapy of infections caused by *P. aeruginosa* strains showing resistance to available antibiotics. *P. aeruginosa* strains are common components of the natural microflora (water of natural reservoirs, soil). In some conditions they can exhibit pathogenic activity. They are often found in wound infections of various origins and localizations, in the lungs of patients with cystic fibrosis or chronic obstructive pulmonary disease, and in individuals with systemic lupus erythematosus.

Phage phiKZ was isolated from the sputum of a patient with chronic pulmonary infection [1] and attracted attention with its unusual type of plaque. On the lawn of *P. aeruginosa* (strain PAO1), the plaques of this phage had pronounced opalescence (a common sign of a high concentration of mature phage particles). A similar trait was found and its cause described for T-even bacteriophages of *E. coli* [37]. This mechanism of lysis inhibition after reinfection could be considered as an ancient and quite common evolutionary trait which can be manifested in phages of different species and in different phage–bacteria systems. Indeed, in the case of *P. aeruginosa* cells infected with phage phiKZ, the lysis inhibition process is genetically controlled. This has been confirmed by the isolation of mutants in different genes that have lost the ability to manifest the lysis inhibition effect and produced plaques devoid of opalescence [38]. However, in the case of phage phiKZ the mechanism of this process is not yet completely clear.

A significant discrepancy between the yield from a single infected bacterium in the experiment of a one-step growth cycle and the production of a large number of phage particles in confluent lysis on Petri dishes (up to 10^12^ phage phiKZ particles per a Petri dish) drew attention for the further study of this phage.

## 3. Structure of the phiKZ Phage Particle

**Figure 1:**
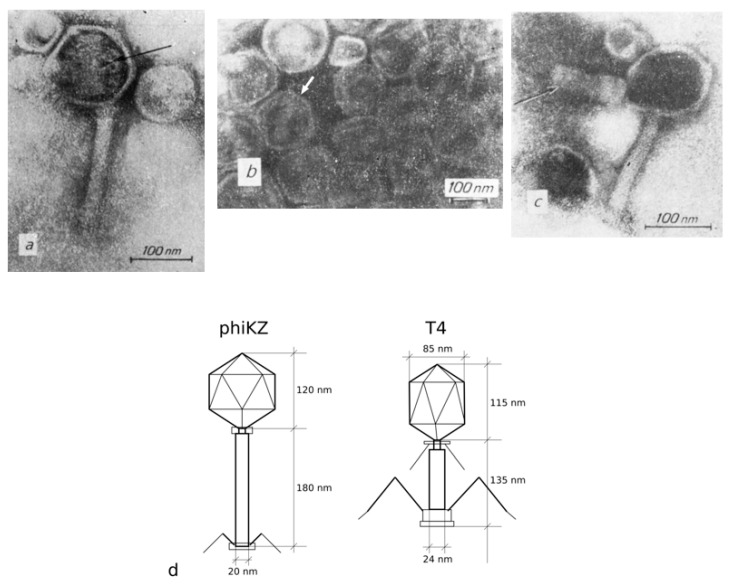
Ultrastructure of phiKZ phage particles after a freezing and thawing procedure: (**a**) Phage particle with a disrupted head. The arrow indicates the inner cylindrical body with loops of fibrous material (perhaps DNA) around; (**b**) The head of a tail-less phiKZ particle with an inner body (the arrow indicates the inner cylindrical body); (**c**) The inner body coming out of the phiKZ particle disrupted by freezing and thawing (arrow) [23]; (**d**) A schematic drawing of phage phiKZ and T4-particles for size comparison.

The first electron micro-graphs of phiKZ phage particles confirmed the large-sized heads of a hexagonal shape to be about 120 nm in size, with a contractile tail measuring 180 × 20 nm in a non-contracted state (Figure 1d). 

Studies of disrupted phage particles that had undergone freezing–thawing procedures allowed, for the first time, the observation of several new details (Figure 1a–c) in DNA packaging inside the phage head. 

One of the images revealed a phage particle with a damaged head. A compact structure designated as the “inner body” was identified inside the particle [1]. Such ordered DNA packaging in phage heads had never been seen before (Figure 1a–c).

The results of experiments on the destruction of phage particles by osmotic shock and electron microscopic observations of phage maturation [19,20] confirmed our assumption for the occurrence of the specific organization of the components of the “inner body” in the phage head (Figure 1c). Thus, as anticipated, the “inner body” consists of a super coiled DNA packed around a cylindrical, spring-like structure that presumably consists of protein.

**Figure 2:**
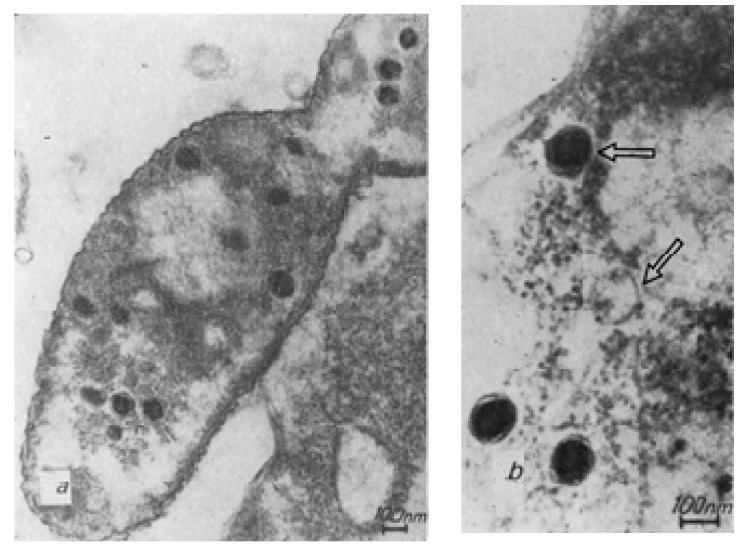
*P. aeruginosa* bacterium (ultrathin section), 120 min after phiKZ infection. (**a**) A whole cell section of *P. aeruginosa;* (**b**) *P. aeruginosa* cell section fragment. Phage heads may represent different stages in phiKZ morphogenesis and DNA compaction (see arrows) [23].

The maturation of the phiKZ bacteriophage in infected *P. aeruginosa* bacteria until the appearance of the first visually distinguishable phage particles has been detected in the study of ultrathin sections of bacteria infected with the phiKZ phage at different times after infection. Successive stages of morphogenesis are observed in the structure of the phage phiKZ particles after infection (Figure 2).

During the initial infection of a bacterium with a phiKZ phage particle at 37 °C, structures that are clearly related to the synthesis of new phage particles appear in cells fixed in the twelfth minute. It is assumed that these may be the initial stages of phage DNA condensation. In empty capsids, a central cylindrical body or a rod attached to opposite poles of the head can be seen.

The process of phage maturation seems to undergo several successive steps. First, a pool of replicating phage DNA is formed, pushing the bacterial nucleoid to the periphery of the cell. Against the background of this pool of DNA, precursors of phage particles are formed, possibly in the form of a DNA strand wound on a rod. Presumably, this is the future inner body. Around this nucleus the head membrane is assembled. In some images of sections of infected bacteria it is possible to distinguish a spiral structure that resembles the inner body of a mature phage particle [23,24].

Almost 30 years after the first studies of the phiKZ phage structure were performed with the help of new modern methods of electron microscopy, studies on this unique bacteriophage continue. The structure of the phiKZ head and tail was determined using cryo-electron microscopy, and three-dimensional image reconstructions were performed by Fokine et al. [39,40]. In these studies, details on the particle size of phiKZ were refined: ~145 nm diameter icosahedral head and a ~200 nm long contractile tail were found, and the phiKZ tail tube was reported to have 4.5nm inner and 11nm outer diameters. Its baseplate has a rather flat, hexagonal shape, with a diameter of ~80 nm and a thickness of ~35 nm. It was also shown that the genomic DNA is packaged into the head capsid as a spool-like structure; the axis of the spool makes an angle of ~32° with the long axis of the phage particle. A differential mapping procedure, based on the physical principle of protein’s greater sensitivity to radiation damage compared to nucleic acid, allowed Wu et al. to locate the inner body in individual nucleocapsids and then to calculate a three-dimensional reconstruction of the inner body (this being ~24 nm wide, ~105 nm long, and consisting of multiple stacked tiers) [41]. Thomas et al., with the use of mass spectrometry and bioinformatic and biochemical studies, performed a detailed study of the processes leading to the change in the structure of the inner body (presumably due to the active proteolysis by the hypothetical morphogenetic protease of the phiKZ phage) and found detailed information on the complex structure of the phiKZ inner body and the complicated processes occurring during phiKZ head maturation [42]. All of this new information confirms the results of early research into the unusual packaging of phage phiKZ DNA.

## 4. Phage PhiKZ Transduction

In an article by Monson et al. (2011) [43] which was devoted to the study of phage phiPA3 (a new member of the phiKZ-like group), the authors mistakenly reported that “this is the first report of a member of this group of phages acting as a generalized transducer”, when the phenomenon was actually first reported in 1982 for phage phiKZ [22]. It was shown that the phiKZ phage carried out the general transduction of bacterial DNA with an average frequency of transductants for different markers from 7 × 10^−6^ to 1.6 × 10^−9^. Later (2002), the ability for general transduction was shown for several other phiKZ-like phages [28]. This makes it possible to use phages of these groups when it is necessary to transduce large fragments of bacterial DNA in *P. aeruginosa* bacteria. At the same time, the mechanism of general transduction by the phiKZ phage itself, taking into account the specific packaging of phage DNA, has not been studied sufficiently. In particular, it is not known in what state the bacterial DNA exists in the transducing phage particle, and whether it is packed with the formation of an internal body or presented in a different form.

## 5. Bacteriophage phiKZ Genome

The phiKZ genome was sequenced by an international team in the very beginning of the 2000s, becoming the longest phage genome to be sequenced at that time (280,334 bp and 306 ORFs) [2]. Some characteristic features were discovered, including low GC content in contrast with the GC-rich host genome, the presence of several tRNAs, many gene products revealing homology to proteins of different bacterial species or other phages active on different hosts, and even more gene products for which homology and putative function could not be quickly identified. Among these gene products with a known relatively high homology there are nucleotide metabolism enzymes, as well as RNA polymerases, some phage structural proteins, and putative phage endonuclease. However, some functionally important proteins such as the DNA polymerase and some other replication-associated proteins were not rapidly found by amino acid homology, and this was a very odd discovery for such a large and rich phage genome.

These initial phiKZ sequence data became a starting point for many subsequent studies. For example, one known endolysin encoded in genome gp144 (transglycosylase), was obtained through cloning into an expression vector, and its properties and structure were then studied; the protein appeared to be part of a phage particle [44].

An interesting series of studies is devoted to the so-called non-canonical non-virion RNA polymerase which is encoded in the early part of the genome. This is absent from phage particles and is responsible for transcription of late-phage genes [45,46]. While four of the five subunits are homologs of cellular RNA polymerase, the fifth subunit is completely obscure. It was shown, however, that this fifth subunit was not only responsible for promoter recognition, since a four-subunit complex lacks catalytic activity, in contrast with a homologous cellular enzyme.

Recently, it was found that that phage phiKZ encodes a proteinaceous shell that assembles a nucleus-like structure that compartmentalizes proteins and DNA during viral infection [47,48] and in this way segregates its DNA from the immunity mechanisms of its bacterial host (two subtypes of CRISPR—Cas3, Cas9, Cas12a and the restriction enzymes HsdRMS and EcoRI) [49]. Phage-encoded tubulin-like protein is supposed to play a role in positioning this structure in the cell [45,50]

The DNA polymerase was finally found in phiKZ-like phages genomes by way of very thorough bioinformatical analysis and appeared to represent an unexpectedly highly divergent newly discovered branch of the B-family DNA polymerases [51,52,53]. However, despite such a serious sequence divergence, this branch was phylogenetically classified as a sister clade with T4-like phages’ DNA polymerases. The analysis of codon and amino acid usage in the phiKZ genome also confirms some degree of similarity with the T4-like phages [54].

The structural proteome of the phiKZ phage has also been studied [55] as well as the subsystem responsible for host metabolism modification and substitution [56].

## 6. Origin of the Group of Phylogenetically Related Species of *P. aeruginosa* Giant Phages and Their Species Diversity

**Figure 3:**
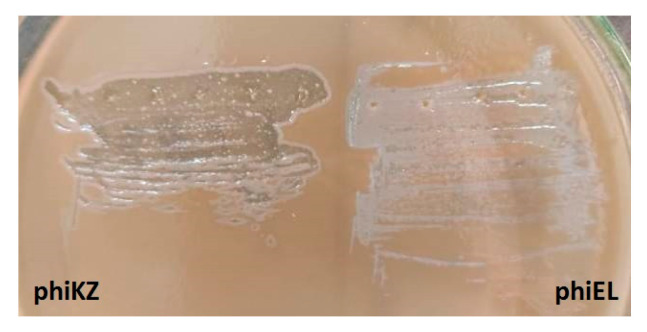
Examples of phiKZ-like phage growth on the lawn of *P. aeruginosa* PAO1 (different degrees of opalescence are visible).

The described feature of the phiKZ bacteriophage—the formation of opalescent plaques (Figure 3)—was used in the search for other species of presumably phylogenetically related giant phages of *P. aeruginosa*. 

Indeed, such phages were found both in samples from the environment (ponds and puddles, river water, and sewage from treatment plants) and in wounds of various origins infected with *P. aeruginosa* bacteria. Several phages have been isolated from the commercial therapeutic multi-species phage mixtures produced by different specialized enterprises. In addition, some phages that we assigned to giant group were found in typing collections provided to us by various authors for study—we are deeply grateful for this help. As a result, 18 giant bacteriophages of different origin (see Table 2) active on *P. aeruginosa* were chosen for further work. 

A detailed study of this group of phages was carried out with regard to electron microscopy, spectra of lytic activity, inactivation with specific antisera, comparisons of DNA restriction and hybridization, comparisons of the polypeptide composition of mature phage particles, and the determination of the N-terminal sequence of the main capsid protein, etc. It turned out that among the group of *P. aeruginosa* giant phages there were both bacteriophages that were similar and related to the phiKZ phage as well as bacteriophages of other species that were phenotypically similar to phiKZ.

As a result, in accordance with the most important criteria for the delineation of the species [60], the giant phages were classified into three species: species phiKZ, species phiLin68, and species phiEL [25,26,27,28,30,38]. It has been proposed that the group of bacteriophages be considered as representative of a new genus among of myovirus bacteriophages (“phiKZ-like viruses”) [61].

**Figure 4:**
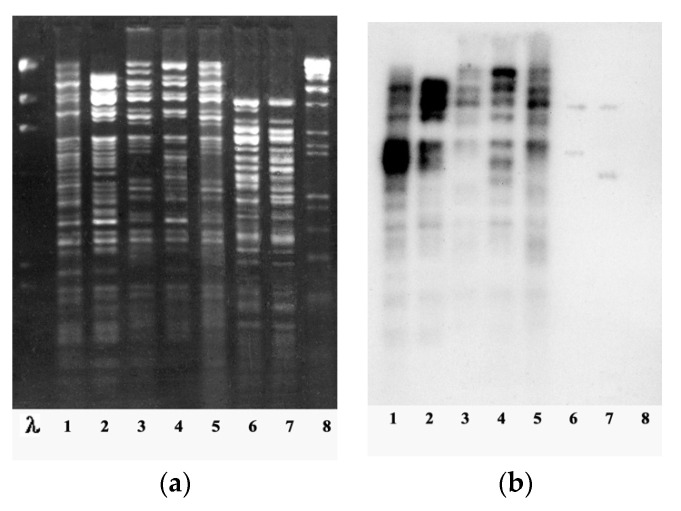
Examples of restriction analysis and DNA hybridization of phiKZ-like phages: (**a**) DNA restriction patterns obtained with *Hin*dIII for bacteriophages: (λ) lambda, (1) phiKZ, (2) phiLBG20, (3) phiLBG21, (4) phiLBG23, (5) phiLBG26, (6) phiLBG22, (7) phiLin68, and (8) phiRu. (**b**) Southern blot hybridization of 32P-labeled phiKZ DNA with *Hin*dIII DNA fragments of phages (1) phiKZ, (2) phiLBG20, (3) phiLBG21, (4) phiLBG23, (5) phiLBG26, (6) phiLBG22, (7) phiLin68, and (8) phiRu [30].

Among the genus of giant “phiKZ-like viruses” of *P. aeruginosa*, the phiKZ species is the most widely represented (13 out of 18 bacteriophages isolated at that time). All bacteriophages of the the phiKZ species had a similar size of phage particles, were inactivated by anti-phiKZ serum, and had a genome sizes similar to that of phiKZ (more than 270 kb). The DNAs of phages in these species had unique restriction profiles and a high level of homology with phage phiKZ, with evident signs of similarities (restriction fragments common for all phages of the species accounted for more than a half of the phage genome) (Figure 4).

Moreover, phiKZ-related phages isolated from different geographic regions (Kazakhstan, Russia, Western Europe) showed no substantial differences, which might be expected in the case of long-term evolution in geographical isolation. Their genomes are mosaic ones, suggesting a high rate of genetic exchange within the species (although high mutability cannot also be excluded). Besides, two pairs of phages (phiLBG21 and phiLBG26, phiPBD1 and phiPBD2) were isolated from different sources but were undistinguishable by several tests and differed only in growth on some clinical strains. The genomes of these phages possibly differ by point mutations. This demonstrates that phages, even those with a high recombination frequency under natural conditions, may exist in highly similar forms in different localities. Most of the scenarios for the emergence of new non-lethal mutations in the genomes of phiKZ-related phages that critically affect phage activity have probably already been used. Since mature particles of these phages are completely identical in polypeptide composition and the restriction sites of most of the genome are conserved, it is possible to assume that mutations and genome rearrangements do not affect the structural genes of these phages [62].

The other species of *P. aeruginosa* giant phages, species Lin68, is represented by only two bacteriophages: Lin68 from the typing collection of Lindberg and the natural phage LBG22. These two phages have a characteristic feature of particle morphology: a shortened tail (160 nm). This in contrast to the rest of the group of *P. aeruginosa* giant phages (180 nm) [26]. Both phages are inactivated by anti-phiKZ serum, but with significantly less efficiency than representatives of the phiKZ species. The DNA of the phiLin68 and phiLBG22 have a similar restriction profile, are homologous to each other, and have a very weak level of homology with the DNA of phiKZ-like phages [30]. These data indicate a direct relationship between these species of phages, although their divergence apparently occurred a long time ago. It is possible that the preserved DNA homology is due to conserved sequences such as those found in the genomes of phylogenetically related phages of the T-even group of *E. coli* [63].

The third species of giant phages of *P. aeruginosa*, a species of phi EL-like phages, is represented by three bacteriophages: phiEL, phiRU and phiChe21/5. Phages of this species are significantly less related to phages of the phiKZ and Lin68 species. They are not inactivated with anti-phiKZ serum and differ in the polypeptide composition of mature particles from phages of the other two species. Despite having the same phage particle size as phiKZ (capsid diameter ∼120 nm), the genome of phiEL-like phages is almost one-third smaller (211,215 bp) and has no DNA homology with the genome of phiKZ phages. In spite of this, 81 out of 201 phiEL gene products were found to be homologous to the phiKZ phage proteins, which confirms the phylogenetic relatedness of these phage species [31,55,62].

## 7. Antibacterial Activity of *P. aeruginosa* Giant Phages and Features of Their Use in Phage Therapy

In commercial therapeutic mixtures produced in Russia one can find both various phiKZ-like phages and giant phages of other species [64,65,66]. It is of interest to compare the spectra of their lytic activity to assess the relative value of phages of different species of this group in terms of their ability to lyse both the initial clinical isolates of *P. aeruginosa* and their mutants exhibiting resistance to phages of other species. In addition, clinical isolates of *P. aeruginosa* often carry plasmids that inhibit phage development, which limits the possibilities of phage therapy.

To give a general assessment of the therapeutic activity of different species of giant phages, their activity has been tested against a collection of 70 randomly selected clinical isolates of *P. aeruginosa*. As it turned out, among the giant phages of different species of our collection taken in the experiment, as a rule it was possible to select combinations of phages that exhibited high total lytic activity with respect to most clinical isolates. Phages of the phiEL-like species exhibit the ability to infect no more than half of randomly selected clinical isolates. However, bacterial mutants selected as resistant to phiEL or phiRU usually do not restrict the growth of phages of other species of the giant phage group. The use of other types of *P. aeruginosa* virulent phages, which are usually included in commercial preparations, increases the proportion of lysed clinical *P. aeruginosa* strains by no more than 20% (according to our data) [67].

Verification of the dependence of the growth of different giant phages on the presence of different plasmids of the incompatibility group IncP2 (PMG73, PMG1, PMG35, Rms139, Rms165, Rip64, RPL11, Rms163, PMG53) in the infected bacteria from the collection of Jacoby GA [58,59,68] showed a very high level of interaction selectivity (Table 2). Most of the plasmids had no negative effect on the growth of the phages of the studied group. Just one plasmid—PMG53 (IncP2)—showed an inhibition of growth of phiKZ and other tested giant phages. At the same time, the Rms148 plasmid of the group (IncP7) inhibited the growth of all phages except phiLin68 and phiLBG22 [30]. Obviously, such a selective dependence of phage activity on the presence of plasmids should be taken into account during phage therapy.

The evolution of some large phages of *P. aeruginosa* can be influenced by their ability to overcome the species barriers of infected bacteria, which allows us to consider the possibility of the participation of interspecies migration of phages in the formation of genomes of related bacterial species. Thus, phages phiLin68 and phiLBG22 infect two strains of bacteria of the species *P. fluorescens* biovar IV [28].

A high degree of independence of the phiKZ phage development from cellular processes that are necessary in the development of phages of other groups (including the host transcription apparatus [69]) is not common to all phylogenetically related large phages in this group of species, but may promote interspecies migration and evolutionary interactions.

From the same point of view, a useful feature of the phiKZ phage is the special structure of phage RNA polymerase, which promotes the expression of the phage genome regardless of the state of the pathogenic bacterium [45].

**Figure 5:**
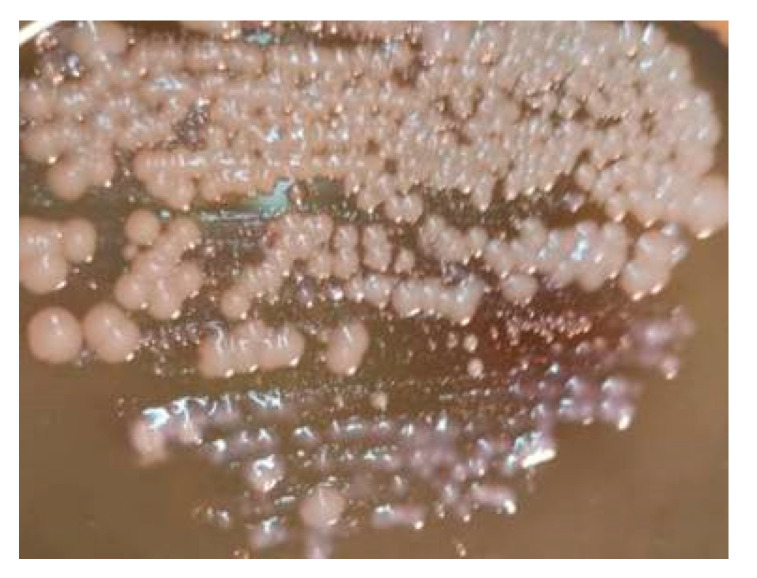
“Pseudolysogenic colonies” after 3 days of incubation—the material seeded from the lysis zone of phage phiKZ after overnight incubation yields many small colonies of bacteria. After further incubation (1–2 days), colonies slightly increase in size and, due to the content of numerous mature phage particles, acquire a blue shade.

As we noted earlier, being virulent, *P. aeruginosa* giant phages are components of several commercial mixtures owing to their broad spectrum of lytic activity. However, it has been shown for the first time that the infection of phiKZ-like phages may convert the bacterial cell to a specific pseudolysogenic state, when the cell continues divisions for several days to form a colony [28]. This state is unstable and such colonies are lysed after a while to yield a great amount of the phage; this process is responsible for the opalescence of negative colonies as an external feature common for *P. aeruginosa* giant phages (Figure 5).

It was shown that the decisive factor in this process is the multiple infection of bacteria with bacteriophages belonging to this group [38]. Such multiple infection causes lysis inhibition without the loss of the cell’s ability to divide and yield colonies (unlike in the case of lysis inhibition in *E. coli* bacteria infected with a phage from the T-even group, when bacteria lose cell dividing ability due to the disruption of the bacterial chromosome [70]). The detection of giant phages in clinical *P. aeruginosa* isolates supports the idea that pseudolysogeny with these phages arises and is maintained in natural conditions. This feature of giant phages can create a number of problems when used in phage therapy: the capacity of phages for pseudolysogeny leads to an increase in the probability of selection during therapy for phage-resistant mutants and genetic exchange with the bacterial chromosome, as well as the release of a large amount of high-polymer DNA during lysis of bacteria, creating significant viscosity and preventing access of other phages to bacteria.

For the successful use of giant phages in therapy, a further study of phages of the phiEL species (phiEL, phiRU, and phiCHE) may be of particular interest. When bacteria are co-infected with these phages, recombinant phages with unusual morphology of negative colonies are found in the offspring with a high frequency [71]. The reason for this may be the accumulation of changes in the genomes of these phages, aimed at adapting to survival in special conditions of infection [65].

As an example, the phiCHE phage isolated from the clinical *P. aeruginosa* strain in deep burn infection was initially considered a temperate phage species. Traces of phiCHE activity during sowing on bacterial lawns can only be detected after several days of incubation. Apparently, this feature reflects the emergence in this phage of a developmental variant intermediate between virulent and moderate modes (possibly as a consequence of the lysis inhibition effect, which is especially pronounced in this phage). Further specially designed experiments are needed to understand how this special state arises in the absence of stable (inherited) lysogenization, when the bacteriophage genome can remain in an infected bacterium for a long time, both without explicit expression of lytic functions and without stable lysogenization.

**Figure 6:**
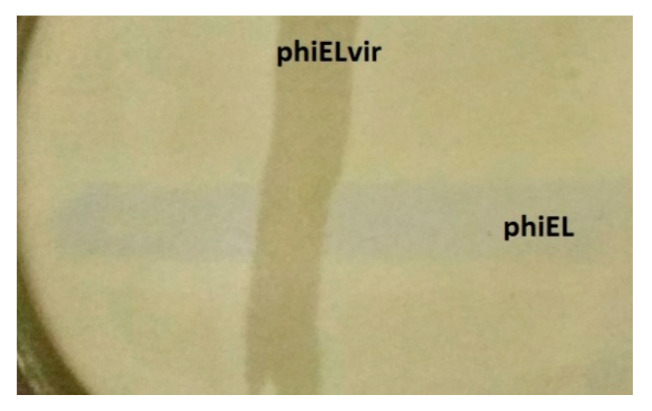
Growth of a virulent mutant phiElvir (no opalescence) over a wild variant of phage phiEL (opalescence). In the place of joint infection, opalescence has disappeared.

Several mutants of various phages belonging to species EL, manifesting the loss of the ability to exist in the pseudolysogenic state, have been isolated. Moreover, such mutants were able to lyse bacteria in a pseudolysogenic state (infected with the corresponding wild-type phages) [38] (Figure 6).

The behavior of such mutants is similar to that of virulent mutants of temperate phages; therefore, we termed the mutants “vir”. We suppose it is expedient to use virulent mutants of phages belonging to this genus rather than phages of the wild type.

It should be noted that not only during phage therapy but during laboratory work with phages in general it is desirable to avoid the creation and active reproduction of mixtures of phages of certain species, for example, giant phages (like phiKZ) and transposable phages (as D3112). It is possible that some specific interactions in the course of joint development may lead, under certain experimental conditions, to unpredictable results, for example, to the emergence of new pathogens with unusually aggressive properties. This can also be assumed on the basis of the detection in the genome of the phiKZ phage of many genes, the gene products of which exhibit obvious similarities with the proteins of pathogenic microorganisms of species such as *Mycobacterium tuberculosis*, *Haemophilus influenzae*, *Listeria sp.*, *Rickettsia prowazakeri*, and *Vibrio cholerae* [2]. This is hardly accidental and most likely reflects the stages of the previous evolution of this phage. On the other hand, it is possible that some of these products may perform a structural function in the formation of a phage particle [30]. The presence of genes with unclear functions in phiKZ and its similarities with potential toxins or proteins of pathogenic bacteria, as well as the ability for general transduction (see below), do not exclude the possibility of direct horizontal transfer of genes of different organisms into the genomes of phages of this group, and their ability for genetic exchange with different hosts. Obviously, further research is needed to understand the possibility of the influence of phiKZ phages on the manifestation of pathogenicity and virulence of infected bacteria [72].

## 8. New Giant Phages of *Pseudomonas aeruginosa* and Other Bacterial Species

It can be considered that one of the most important consequences of the discovery of the phiKZ phage is the emergence of a kind of informal competition among researchers, which continues to this day, in the search for unusually large phages with the hope of finding unusual properties in them. Such hopes are often justified. The review from Yuan and Gao (2017) [73] refers to the results of research and genome sequencing of approximately 50 giant phages. Over the past 3 years, the number of sequenced genomes in the NCBI database has doubled. This indicates the growing interest of the scientific community in the study of this group of phages. In Table S1 we present giant bacteriophages active on different bacterial species with a sequenced complete genome. The currently described giant bacteriophages of various bacterial species (excluding *P. aeruginosa*) are important for understanding the evolution and morphogenesis of these phages as well as phage–host interactions. In addition, these studies are one of the elements for studying the possibility of practical application of giant phages in medicine, veterinary science, and agriculture. Here, we would like to mention just a few of them.

As an example, there are interesting studies on the phage *Erwinia amylovora* vB_EamM_Y3, which is phylogenetically related to the phages of *P. aeruginosa* PaBG and phage *P. putida* Lu11 and is distantly related to the phage *Ralstonia solanacearum* ΦRSL1. Together, they represent a distinct lineage of hairy myoviruses [74,75,76]. The inclusion of new giant bacteriophages into the study leads to discoveries of unexpected relationships between phages of distant bacterial species. Thus, work on the assembly of the genome of a giant (257,908-bp) bacteriophage called NCTB (New Caledonia) of the cyanobacterium *Trichodesmium* bloom [77] has led to the discovery of its unexpected kinship with the *P. aeruginosa* phage PaBG from Lake Baikal and the Lu11 phage from a soil sample from the Philippines. Another example of an unexpected similarity has been found in the packaging of the genomes of phages of unrelated bacteria: the presence of an internal body in phage vB_KleM-RaK2, active on *Klebsiella* [78].

Studies of host–phage interactions can be illustrated by a publication devoted to giant phages of oceanic cyanobacteria *Prochlorococcus*; these cyanophages contain genes that reflect adaptations for infection of photosynthetic hosts in oceanic environments with low nutrient content. These are the photosynthetic genes (psbA, hliP), which are thought to help maintain the photosynthetic activity of the host during infection, and the gene for the aldolase family (talC), which may facilitate alternative pathways for carbon metabolism during infection [79].

Some giant phages are used in phage therapy mixtures and for agricultural purposes. For example, to test the potential of the phage CR5 as a biocontrol agent against *Cronobacter sakazakii*, it was added to infant formula contaminated with *C. sakazakii* clinical isolate or to food isolate, and showed complete inhibition of bacterial growth [80]. Effective phage cocktails containing giant phages AS-szw, AS-yj, AS-zj, AS-sw, and AS-gz have been proposed against *Aeromonas salmonicida* infections in fish [81]. The giant phage *P. fluorescence* OBP [34] is of considerable interest both from a therapeutic and scientific point of view: in the absence of DNA homology with phiKZ, it has good amino acid similarity. An internal body was also found inside it, which is a distinctive feature for all phages of this group [82]. The OBP genome encodes the GroEL-like chaperonin, the structure participating in the refolding of large proteins. Due to its unique structural and functional features, OBP chaperonin can represent a new group [83,84]. Its study is important for elucidating the positive and negative role of chaperonines in neurodegenerative diseases caused by the formation of amyloid structures [85,86]. The other giant *Pseudomonas* phage PA5oct, with genome of 375 kbp, has been isolated from a sample taken from an irrigated field [87]. It can now be considered as the interim champion in this genome size competition, significantly outperforming the next in size—phage GAP32, active on Gram-negative *Cronobacter sakazakii* (genome size 358,663bp) [88]. As *Cronobacter sakazakii* is able to cause meningitis in small children [89], the giant phiGAP32 can be considered as potentially useful for phage therapy. The development of research on the role of bacteriophages in the production of agricultural products, including their use as a means of combating bacteria pathogenic for agricultural crops, can provide a source of new ideas, useful for expanding the possibility of safe phage therapy in the treatment of infectious diseases in humans and animals. It is also important that these phages are safer models (in comparison with phages of bacteria pathogenic for humans) for studying subtle molecular interactions [90,91,92].

## 9. The Future of PhiKZ-like Phages—Basic Science and Practical Application

The bacteriophage phiKZ has been studied for 40 years. However, these studies remain relevant both for fundamental science and for the applied purposes of phage therapy.

Modern technologies provide perspectives for in depth studies and new discoveries. Advances in the use of cryo-electron microscopy, cryo-electron tomography, atomic force microscopy, etc. have allowed for breakthrough discoveries in the fine structure of phage particles and molecular interactions. The possibilities for genomic analysis and the availability of sequencing methods may bring phylogenetics to a high level. The application of these approaches will undoubtedly make it possible to find answers to important questions concerning the infectious process and mechanisms of gene regulation, evolution, and the origin of giant phages.

New knowledge acquired on the topic of giant phages will be in demand for practical applications including phage therapy: many already discovered giant phages are active on pathogenic bacteria. It is widely known that the multiple resistance of pathogenic microorganisms to antibiotics has become one of the most important problems in world health [93,94]. Under these conditions, the experience of using bacteriophages as antibacterial agents and the study of new possibilities of phage therapy have become especially relevant [95,96,97]. In bacteriophage therapy, both live phages and phage engineering products can be used [98,99].

phiKZ-like bacteriophages are active on the bacteria *P. aeruginosa*, which are dangerous due to their ability to quickly acquire resistance to most known antibiotics and to form biofilms in inflammatory foci [100,101]. Due to the unusually wide spectrum of lytic activity, the ease of obtaining them in high titers, their resistance to treatment with various agents, and good storage stability, phiKZ-like phages are considered promising in terms of phage therapy and are often used in phage therapy mixtures [65,66,102,103]. Some phiKZ-like phages are even capable, at least in vitro, of destroying biofilms of *P. aeruginosa* strains [104,105]. Moreover, the application of phiKZ-like phages (phiKZ, phiEL, and OBP) lysins to combat multidrug resistant *P. aeruginosa* strains has been reported [106,107,108]. In connection with a number of features of phiKZ-like bacteriophages, a problem arises with regard to the precautions that need to be taken when using them for phage therapy, but we believe that the potential of this group of phages has not yet been fully revealed.

As is often the case, the more we learn, the more questions we have. We consider it very important to continue further in-depth studies on giant phages and their interaction with host bacteria and other bacteriophages, and search for new giant phages from natural sources. We are confident that subsequent studies will reveal new, as yet unstudied species of giant phages *P. aeruginosa* and other bacterial species.

## Figures and Tables

**Table 1:** Main steps in the early research history of giant phages.

Years	Scientific Research
**1978**	- First description of the structure and some biological properties of the new virulent *Pseudomonas* phage phiKZ with unique features distinguishing this phage from all those known before, including a large size of the head and DNA, and the presence in the head of an unusual complicated structural formation of a cylindrical shape named the “central or inner body” [1,19].
**1980–1982**	- The results, obtained with tailless mutants of phage phiKZ, allowed for the circular location of the fibrous material around the “inner body” to be proven. It was shown that the genetic map of phiKZ is circular, while the dsDNA has a block structure, and the GC pair content is equal to 44% [20,21]. - It was shown that phiKZ is a general transducing phage [22].
**1983–1984**	- A detailed electron microscopical examination of phage phiKZ confirmed that in the center of the phage head is a cylinder of low electron density (“inner body”) coated by fibrinous material which is packaged around the inner body in a spool-like manner. These structures disappear after the adsorption of phage particles in bacteria. On studying the maturation of the phiKZ bacteriophage in infected *Pseudomonas aeruginosa* bacteria, successive stages of changes in the structure of phage particles after infection were observed. The mechanism for the packaging and ejection of phiKZ DNA was proposed [23,24].
**1985**	- It was shown that the Lin21 (later known as phiLin21) bacteriophage (from the Lindbergh collection, with a large phage particle), contains in its capsid a central body similar in structure to that of phiKZ and is related to phage phiKZ. It was considered that the phages were representatives of a group of related phiKZ-like phages [25].
**1992–1993**	- It was shown that bacteriophage Lin68 (later known as phiLin68) forms opalescent negative colonies (typical of phiKZ-related phages) similar to phage phiKZ in genome size and morphology of phage particle, but with a shorter tail. Phage Lin68 was assigned to another morphotype and another species [26,27].
**2002**	- The complete nucleotide sequence of phiKZ DNA and a preliminary analysis of its genome structure were presented. The genome contains 280,334 bp, and has 306 ORFs and 6 tRNAs. It was the largest bacteriophage genome to be fully sequenced at that time [2] - A comparative study was made of a group of *P. aeruginosa* virulent giant DNA bacteriophages similar to phage phiKZ with regard to several genetic and phenotypic properties. For all phages a broad spectrum of lytic activity was shown, as well as the ability to overcome the suppressing effect of plasmids and the capability for general transduction and pseudolysogeny. By DNA homology the phages were assigned to three species (represented by phages phiKZ, Lin68, and EL (later known as phiEL), respectively) and two new genera (phiKZ and EL) [28]. - For the first time, the term “giant phages” was used to denote a group of bacteriophages similar to phiKZ (with unusually large capsids and genome sizes).
**2003**	- The complete genome sequence of the T4-like broad-host-range vibriophage KVP40 was determined. The KVP40 genome is 244,835 bp in size and is related to the phage T4 genome [29].
**2004**	- A study of nine phiKZ-like giant phages isolated from natural sources was carried out. It was concluded that phages of the phiKZ species were common in natural populations of various regions, while EL- and Lin68-related phages were extremely rare. Phages isolated in different geographical regions showed no substantial differences [30].
**2005**	- The complete nucleotide sequence of second phiKZ-like giant phage EL was presented. The EL genome comprises 211,215 bp and has 201 predicted ORFs. The EL genome does not share its DNA sequence homology with any other viruses and micro-organisms sequenced to date. However, one-third of the predicted EL gene products share a similarity with phiKZ proteins [31].
**2005–2008**	- New data on the isolation and study of new giant bacteriophages active on different species and genera of bacteria appeared with regard to phiSMA5 (*Stenotrophomonas maltophilia*) [32], piR1-37 (*Yersinia enterocolitica*) [33], Lu11 (*P. putida* var. Manila) and OBP (*P. fluorescens*) [34], 201varphi2-1 (*P. chlororaphis*) [35], and 0305phi8-36 (*Bacillus thuringiensis*) [36].
**2009**	- For tailed bacteriophages with genomes of more than 200 kbp of DNA the term “jumbo phages” was proposed [8].
**Since 2010**	- Mass sequencing of bacteriophage genomes began, with detection of phages with a large genome size active on different bacteria species. Studies on the possibilities of using giant bacteriophages for antibacterial therapy have been carried out.

**Table 2:** Properties of *Pseudomonas aeruginosa* phiKZ-like phage species.

Name	Source And Geographical Location Of The Phage Isolation	Capsid Size (D, nm)	Tail size (L/W, nm)	Genome Size, bp; (NCBI Reference Sequence, if Available)	Growth on Plasmid-Containing *P. aeruginosa*		
					PAO303 (Rms148) *	PAO38 (PMG53) *	PAO1 Mutants with Plasmids * Rms165, Rpl11, Rip64, Pmg73, Rms139, Pmg1, Pmg35, Rms149, Rms163
**phages of the phiKZ species**							
phiKZ	sputum of a patient with pulmonary infection (Kazakhstan)	120	180/20	280,334; (NC_004629.1)	-	-	+
phiLin21	Lindberg *Pseudomonas aeruginosa* typing phage collection [57] were kindly transferred us by Prof. H-W. Ackermann, (Canada)	120	180/20	~290,000	-	-	+
phiLBG20 phiLBG21 phiLBG23 phiLBG26	water sources of Moscow and Moscow region (Russia)						
phiPBD1 phiPBD2 phiPBD3 phiPBD4	Danube and its tributary (Germany)						
PTB80	commercial phage mix produced in Tbilisi (Georgia)						
phiNN	commercial phage mix produced in Nizhnii Novgorod (Russia)						
phiChe2/2 **	infected post-burn wound of patient in Burn Department of Chelyabinsk hospital (Russia)						
**phages of the Lin68 species**							
phiLin68	Lindberg *Pseudomonas aeruginosa* typing phage collection [26] were kindly transferred us by Prof. H-W. Ackermann, (Canada)	120	160/20	~290,000	+	-	+
phiLBG22	water sources of Moscow region (Russia)						
**phages of the EL species**							
phiEL	water sources of Moscow region (Russia)	120	180/20	211,215 (NC_007623.1)	-	-	+
phiRU	water sources of Moscow region (Russia)	120	180/20	~220,000	-	-	+
phiChe21/5 (later named as phiCHE) **	infected post-burn wound of patient in Burn Department of Chelyabinsk hospital (Russia)						

* All plasmids from the collection of Dr. Jacoby G.A. (USA) [58,59]; ** Not tested in plasmid-dependent growth experiment.

## Data Availability

This review uses data from published scientific articles only.

## Supplementary Materials

The following are available online at https://www.mdpi.com/1999-4915/13/2/149/s1, Table S1: Giant phages active on different bacterial species.

## References

[1] Krylov V.N., Zhazykov I. (1978). Pseudomonas bacteriophage phiKZ-possible model for studying the genetic control of morphogenesis. Genetika.

[2] Mesyanzhinov V.V., Robben J., Grymonprez B., Kostyuchenko V.A., Bourkaltseva M.V., Sykilinda N.N., Krylov V.N., Volckaert G. (2002). The genome of bacteriophage φKZ of Pseudomonas aeruginosa. J. Mol. Biol..

[3] Bradley D.E. (1963). The Structure of Coliphages. J. Gen. Microbiol..

[4] Yap M.L., Rossmann M.G. (2014). Structure and function of bacteriophage T4. Futur. Microbiol..

[5] Ackermann H.-W. (2012). Bacteriophage Electron Microscopy. Adv. Clin. Chem..

[6] Forterre P. (2017). Viruses in the 21st Century: From the Curiosity-Driven Discovery of Giant Viruses to New Concepts and Definition of Life. Clin. Infect. Dis..

[7] Brandes N., Linial M. (2019). Giant Viruses—Big Surprises. Viruses.

[8] Hendrix R.W. (2009). Jumbo Bacteriophages. Curr. Top. Microbiol. Immun..

[9] Cornelissen A., Hardies S.C., Shaburova O.V., Krylov V.N., Mattheus W., Kropinski A.M., Lavigne R. (2011). Complete Genome Sequence of the Giant Virus OBP and Comparative Genome Analysis of the Diverse KZ-Related Phages. J. Virol..

[10] Brown J.M., LaBarre B.A., Hewson I. (2013). Characterization of Trichodesmium-associated viral communities in the eastern Gulf of Mexico. FEMS Microbiol. Ecol.

[11] Luo Z.-H., Yu Y.-P., Jost G., Xu W., Huang X.-L. (2015). Complete genome sequence of a giant Vibrio bacteriophage VH7D. Mar. Genom..

[12] Kim S.G., Jun J.W., Giri S.S., Yun S., Kim H.J., Kang J.W., Han S.J., Jeong D., Park S.C. (2019). Isolation and characterisation of pVa-21, a giant bacteriophage with anti-biofilm potential against Vibrio alginolyticus. Sci. Rep..

[13] Kim S.G., Giri S.S., Yun S., Kim H.J., Kang J.W., Han S.J., Kwon J., Jun J.W., Oh W.T., Park S.C. (2019). Genomic characterization of bacteriophage pEt-SU, a novel phiKZ-related virus infecting Edwardsiella tarda. Arch. Virol..

[14] Weintraub S.T., Redzuan N.H.M., Barton M.K., Amin N.A.M., Desmond M.I., Adams L.E., Ali B., Pardo S., Molleur D., Wu W. (2018). Global Proteomic Profiling ofSalmonellaInfection by a Giant Phage. J. Virol..

[15] Wilson K., Ely B. (2019). Analyses of four new Caulobacter Phicbkviruses indicate independent lineages. J. Gen. Virol..

[16] Lavysh D., Sokolova M.L., Minakhin L., Yakunina M., Artamonova T.O., Kozyavkin S., Makarova K.S., Koonin E.V., Severinov K. (2016). The genome of AR9, a giant transducing Bacillus phage encoding two multisubunit RNA polymerases. Virology.

[17] Kurochkina L.P., Semenyuk P.I., Sykilinda N.N., Miroshnikov K.A. (2018). The unique two-component tail sheath of giant Pseudomonas phage PaBG. Virology.

[18] Khoa H.V., Midorikawa Y., Uchino T., Nakai T., Kato G., Kondo H., Hirono I., Labaiden M., Direkbusarakom S., Sano M. (2017). Complete Genome Sequence of the Lytic Giant Bacteriophage pT24 Infecting Tenacibaculum spp., Isolated from a Shrimp Culture Pond. Genome Announc..

[19] Krylov V.N., Smirnova T.A., Rebentish B.A., Minenkova I.B. (1978). Stucture of PhiKZ bacteriophage particles. Vopr. Virusol..

[20] Tiaglov B.V., Krylov V.N., Plotnikova T.G., Minaev V.E., Permogorov V.I. (1980). Certain physico-chemical properties of bacteriophage phiKZ. Mol. Biol..

[21] Plotnikova T.G., Dzhusupova A.B., Khrenova E.A., Krylov V.N. (1982). Genetic and pheno-genetic studies of the group of ts-mutants of Pseudomonas aeruginosa PAO1 phiKZ phage. Genetika.

[22] Dzhusupova A.B., Plotnikova T.G., Krylov V.N. (1982). Detection of transduction by virulent bacteriophage phi KZ of Pseudomo-nas aeruginosa chromosomal markers in the presence of plasmid RMS148. Genetika.

[23] Smirnova T.A., Minenkova I.B., Khrenova E.A., Plotnikova T.G., Krylov V.N. (1983). Electron microscope study of the intracel-lular development of the Pseudomonas aeruginosa bacteriophage phi KZ. Zhurnal Mikrobiol. Epidemiol. Immunobiol..

[24] Krylov V.N., Smirnova T.A., Minenkova I.B., Plotnikova T.G., Zhazikov I.Z., Khrenova E.A. (1984). Pseudomonas bacteriophage contains an inner body in its capsid. Can. J. Microbiol..

[25] Khrenova E.A., Akhverdyan V.Z., Krylov V.N. (1985). Relation of Pseudomonas aeruginosa bacteriophages phiKZ and 21 processing the unique nucleoprotein structure in heads. Genetika.

[26] Sharibjanova T.O., Akhverdyan V.Z., Krylov V.N. (1992). A comparative study of DNA homology and morphology of Pseudomonas aeruginosa bacteriophages to reveal phylogenetic relationships and for an express-classification. Genetika.

[27] Krylov V.N., Tolmachova T.O., Akhverdian V.Z. (1993). DNA homology in species of bacteriophages active on Pseudomonas aeruginosa. Arch. Virol..

[28] Burkal’tseva M.V., Krylov V.N., Pleteneva E.A., Shaburova O.V., Krylov S.V., Volkart G., Sykilinda N.N., Kurochkina L.P., Mesianzhinov V.V. (2002). Phenogenetic characterization of a group of giant Phi KZ-like bacteriophages of Pseudomonas aeruginosa. Russ. J. Genet..

[29] Miller E.S., Heidelberg J.F., Eisen J.A., Nelson W.C., Durkin A.S., Ciecko A., Feldblyum T.V., White O., Paulsen I.T., Nierman W.C. (2003). Complete Genome Sequence of the Broad-Host-Range Vibriophage KVP40: Comparative Genomics of a T4-Related Bacteriophage. J. Bacteriol..

[30] Krylov V.N., Bourkaltseva M.V., Sykilinda N.N., Pleteneva E.A., Shaburova O.V., Kadykov V.A., Miller S., Biebl M. (2004). Comparisons of the Genomes of New Giant Phages Isolated from Environmental Pseudomonas aeruginosa Strains of Different Regions. Russ. J. Genet..

[31] Hertveldt K., Lavigne R., Pleteneva E., Sernova N., Kurochkina L., Korchevskii R., Robben J., Mesyanzhinov V., Krylov V.N., Volckaert G. (2005). Genome Comparison of Pseudomonas aeruginosa Large Phages. J. Mol. Biol..

[32] Chang H.-C., Chen C.-R., Lin J.-W., Shen G.-H., Chang K.-M., Tseng Y.-H., Weng S.-F. (2005). Isolation and Characterization of Novel Giant Stenotrophomonas maltophilia Phage φSMA5. Appl. Environ. Microbiol..

[33] Kiljunen S., Hakala K., Pinta E., Huttunen S., Pluta P., Gador A., Lönnberg H., Skurnik M. (2005). Yersiniophage ϕR1-37 is a tailed bacteriophage having a 270 kb DNA genome with thymidine replaced by deoxyuridine. Microbiology.

[34] Shaburova O.V., Hertveldt K., de la Crus D.M., Krylov S.V., Pleteneva E.A., Burkaltseva M.V., Lavigne R., Volcaert G., Krylov V.N. (2006). Comparison of new giant bacteriophages OBP and Lu11 of soil pseudomonads with bacteriophages of phiKZ-supergroup of Pseudomonas aeruginosa. Genetika.

[35] Thomas J.A., Rolando M.R., Carroll C.A., Shen P.S., Belnap D.M., Weintraub S.T., Serwer P., Hardies S.C. (2008). Characterization of Pseudomonas chlororaphis myovirus 201ϕ2-1 via genomic sequencing, mass spectrometry, and electron microscopy. Virology.

[36] Serwer P., Hayes S.J., Thomas J.A., Hardies S.C. (2007). Propagating the missing bacteriophages: A large bacteriophage in a new class. Virol. J..

[37] Doermann A.H. (1948). Lysis and Lysis Inhibition with Escherichia coli Bacteriophage. J. Bacteriol..

[38] Pleteneva E.A., Krylov S.V., Shaburova O.V., Bourkal’Tseva M.V., Miroshnikov K.A., Krylov V.N. (2010). Pseudolysogeny of Pseudomonas aeruginosa bacteria infected with φKZ-like bacteriophages. Russ. J. Genet..

[39] Fokine A., Kostyuchenko V.A., Efimov A.V., Kurochkina L.P., Sykilinda N.N., Robben J., Volckaert G., Hoenger A., Chipman P.R., Battisti A.J. (2005). A Three-dimensional Cryo-electron Microscopy Structure of the Bacteriophage ϕKZ Head. J. Mol. Biol..

[40] Fokine A., Battisti A.J., Bowman V.D., Efimov A.V., Kurochkina L.P., Chipman P.R., Mesyanzhinov V.V., Rossmann M.G. (2007). Cryo-EM Study of the Pseudomonas Bacteriophage φKZ. Structure.

[41] Wu W., Thomas J.A., Cheng N., Black L., Steven A. (2012). Bubblegrams Reveal the Inner Body of Bacteriophage ϕKZ. Science.

[42] Thomas J.A., Weintraub S.T., Wu W., Winkler D.C., Cheng N., Steven A.C., Black L.W. (2012). Extensive proteolysis of head and inner body proteins by a morphogenetic protease in the giantPseudomonas aeruginosaphage φKZ. Mol. Microbiol..

[43] Monson R., Foulds I., Foweraker J., Welch M., Salmond G.P.C. (2011). The Pseudomonas aeruginosa generalized transducing phage φPA3 is a new member of the φKZ-like group of ‘jumbo’ phages, and infects model laboratory strains and clinical isolates from cystic fibrosis patients. Microbiology.

[44] Miroshnikov K.A., Faizullina N.M., Sykilinda N.N., Mesyanzhinov V.V. (2006). Properties of the endolytic transglycosylase encoded by gene 144 of Pseudomonas aeruginosa bacteriophage phiKZ. Biochemistry (Moscow).

[45] Yakunina M., Artamonova T., Borukhov S., Makarova K.S., Severinov K., Minakhin L. (2015). A non-canonical multisubunit RNA polymerase encoded by a giant bacteriophage. Nucleic Acids Res..

[46] Orekhova M., Koreshova A., Artamonova T.O., Khodorkovskii M., Yakunina M.V. (2019). The study of the phiKZ phage non-canonical non-virion RNA polymerase. Biochem. Biophys. Res. Commun..

[47] Chaikeeratisak V., Nguyen K., Egan M.E., Erb M.L., Vavilina A., Pogliano J. (2017). The Phage Nucleus and Tubulin Spindle Are Conserved among Large Pseudomonas Phages. Cell Rep..

[48] Danilova Y.A., Belousova V.V., Moiseenko A.V., Vishnyakov I.E., Yakunina M.V., Sokolova O.S. (2020). Maturation of Pseudo-Nucleus Compartment in *P. aeruginosa*, Infected with Giant phiKZ Phage. Viruses.

[49] Mendoza S.D., Nieweglowska E.S., Govindarajan S., Leon L.M., Berry J.D., Tiwari A., Chaikeeratisak V., Pogliano J., Agard D.A., Bondy-Denomy J. (2020). A bacteriophage nucleus-like compartment shields DNA from CRISPR nucleases. Nat. Cell Biol..

[50] Aylett C.H., Izoré T., Amos L.A., Löwe J. (2013). Structure of the Tubulin/FtsZ-Like Protein TubZ from Pseudomonas Bacteriophage ΦKZ. J. Mol. Biol..

[51] Kazlauskas D., Venclovas Č. (2011). Computational analysis of DNA replicases in double-stranded DNA viruses: Relationship with the genome size. Nucleic Acids Res..

[52] Kazlauskas D., Krupovic M., Venclovas Č. (2016). The logic of DNA replication in double-stranded DNA viruses: Insights from global analysis of viral genomes. Nucleic Acids Res..

[53] Kazlauskas D., Krupovic M., Guglielmini J., Forterre P., Venclovas Č. (2020). Diversity and evolution of B-family DNA polymerases. Nucleic Acids Res..

[54] Sau K., Sau S., Mandal S.C., Ghosh T.C. (2005). Factors Influencing the Synonymous Codon and Amino Acid Usage Bias in AT-rich Pseudomonas aeruginosa Phage PhiKZ. Acta Biochim. Biophys. Sin..

[55] Lecoutere E., Ceyssens P.-J., Miroshnikov K.A., Mesyanzhinov V.V., Krylov V.N., Noben J.-P., Robben J., Hertveldt K., Volckaert G., Lavigne R. (2009). Identification and comparative analysis of the structural proteomes of ϕKZ and EL, two giant Pseudomonas aeruginosa bacteriophages. Proteomics.

[56] De Smet J., Zimmermann M., Kogadeeva M., Ceyssens P.-J., Vermaelen W., Blasdel B., Jang H.B., Sauer U., Lavigne R. (2016). High coverage metabolomics analysis reveals phage-specific alterations to Pseudomonas aeruginosa physiology during infection. ISME J..

[57] Lindberg R.B., Latta R.L. (1974). Phage Typing of Pseudomonas aeruginosai Clinical and Epidemiologic Considerations. J. Infect. Dis..

[58] Jacoby G., Sutton L. (1982). Restriction-modification systems determined by Pseudomonas plasmids. Plasmid.

[59] Jacoby G.A., Sutton L. (1991). Properties of plasmids responsible for production of extended-spectrum beta-lactamases. Antimicrob. Agents Chemother..

[60] Ackermann H.-W., Dubow M.S., Jarvis A.W., Jones L.A., Krylov V.N., Maniloff J., Rocourt J., Safferman R.S., Schneider J., Seldin L. (1992). The species concept and its application to tailed phages. Arch. Virol..

[61] Krylov V.N., Cruz D.M.D., Hertveldt K., Ackermann H.-W. (2007). “φKZ-like viruses”, a proposed new genus of myovirus bacteriophages. Arch. Virol..

[62] Krylov V.N., Miroshnikov K.A., Krylov S.V., Veyko V.P., Pleteneva E.A., Shaburova O.V., Bourkal’Tseva M.V. (2010). Interspecies migration and evolution of bacteriophages of the genus phiKZ: The purpose and criteria of the search for new phiKZ-like bacteriophages. Russ. J. Genet..

[63] Tétart F., Desplats C., Kutateladze M., Monod C., Ackermann H.-W., Krisch H.M. (2001). Phylogeny of the Major Head and Tail Genes of the Wide-Ranging T4-Type Bacteriophages. J. Bacteriol..

[64] Krylov V.N. (2001). Phage Therapy in Terms of Bacteriophage Genetics: Hopes, Prospects, Safety, Limitations. Russ. J. Genet..

[65] Krylov V., Shaburova O., Pleteneva E., Krylov S., Kaplan A., Burkaltseva M., Polygach O., Chesnokova E. (2015). Selection of phages and conditions for the safe phage therapy against Pseudomonas aeruginosa infections. Virol. Sin..

[66] Krylov V.N. (2014). Bacteriophages of Pseudomonas aeruginosa. Adv. Clin. Chem..

[67] Pleteneva E.A., Shaburova O.V., Burkaltseva M.V., Krylov S.V., Kaplan A.M., Chesnokova E.N., Polygach O.A., Voroshilova N.N., Mikhailova N.A., Zverev V.V. (2016). Novel approach to composition of, bacteriophage mixtures for antibacterial therapy. J. Microbiol. Epidemiol. Immunobiol..

[68] Jacoby G.A. (2009). AmpC beta-lactamases. Clin. Microbiol. Rev..

[69] Ceyssens P.J., Minakhin L., Van den Bossche A., Yakunina M., Klimuk E., Blasdel B., De Smet J., Noben J.P., Bläsi U., Severinov K. (2014). Development of giant bacteriophage ϕKZ is independent of the host transcription apparatus. J. Virol..

[70] Warren R.J., Bose S.K. (1968). Bacteriophage-induced Inhibition of Host Functions 1. J. Virol..

[71] Krylov S.V., Pleteneva E.A., Bourkaltseva M.V., Shaburova O.V., Miroshnikov K.A., Lavigne R., Cornelissen A., Krylov V.N. (2011). Genome instability of Pseudomonas aeruginosa phages of the EL species: Examination of virulent mutants. Russ. J. Genet..

[72] Krylov V.N. (2003). The role of horizontal gene transfer by bacteriophages in the origin of pathogenic bacteria. Russ. J. Genet..

[73] Yuan Y., Gao M. (2017). Jumbo Bacteriophages: An Overview. Front. Microbiol..

[74] Adriaenssens E.M., Mattheus W., Cornelissen A., Shaburova O., Krylov V.N., Kropinski A.M., Lavigne R. (2012). Complete genome sequence of the giant Pseudomonas phage Lu11. J. Virol..

[75] Sykilinda N.N., Bondar A.A., Gorshkova A.S., Kurochkina L.P., Kulikov E.E., Shneider M.M., Kadykov V.A., Solovjeva N.V., Kabilov M.R., Mesyanzhinov V.V. (2014). Complete Genome Sequence of the Novel Giant Pseudomonas Phage PaBG. Genome Announc..

[76] Buttimer C., Born Y., Lucid A., Loessner M.J., Fieseler L., Coffey A. (2018). Erwinia amylovora phage vB_EamM_Y3 represents another lineage of hairy Myoviridae. Res. Microbiol..

[77] Pfreundt U., Spungin D., Hou S., Voß B., Bermanfrank I., Hess W.R. (2017). Genome of a giant bacteriophage from a decaying Trichodesmium bloom. Mar. Genom..

[78] Simoliūnas E., Kaliniene L., Truncaitė L., Zajančkauskaitė A., Staniulis J., Kaupinis A., Ger M., Valius M., Meškys R. (2013). Klebsiella phage vB_KleM-RaK2—A giant singleton virus of the family Myoviridae. PLoS ONE.

[79] Sullivan M.B., Coleman M.L., Weigele P., Rohwer F., Chisholm S.W. (2005). Three Prochlorococcus Cyanophage Genomes: Signature Features and Ecological Interpretations. PLoS Biol..

[80] Lee J.-H., Bai J., Shin H., Kim Y., Park B., Heu S., Ryu S. (2015). A Novel Bacteriophage Targeting Cronobacter sakazakii Is a Potential Biocontrol Agent in Foods. Appl. Environ. Microbiol..

[81] Chen L., Yuan S., Liu Q., Mai G., Yang J., Deng D., Zhang B., Liu C., Ma Y. (2018). In Vitro Design and Evaluation of Phage Cocktails Against Aeromonas salmonicida. Front. Microbiol..

[82] Sokolova O.S., Shaburova O., Pechnikova E., Shaytan A., Krylov S., Kiselev N., Krylov V.N. (2014). Genome packaging in EL and Lin68, two giant phiKZ-like bacteriophages of P. aeruginosa. Virology.

[83] Semenyuk P.I., Orlov V.N., Sokolova O.S., Kurochkina L.P. (2016). New GroEL-like chaperonin of bacteriophage OBP Pseudomonas fluorescens suppresses thermal protein aggregation in an ATP-dependent manner. Biochem. J..

[84] Stanishneva-Konovalova T.B., Semenyuk P.I., Kurochkina L.P., Pichkur E.B., Vasilyev A.L., Kovalchuk M.V., Kirpichnikov M.P., Sokolova O.S. (2020). Cryo-EM reveals an asymmetry in a novel single-ring viral chaperonin. J. Struct. Biol..

[85] Wälti M.A., Steiner J., Meng F., Chung H.S., Louis J.M., Ghirlando R., Tugarinov V., Nath A., Clore G.M. (2018). Probing the mechanism of inhibition of amyloid-β(1–42)–induced neurotoxicity by the chaperonin GroEL. Proc. Natl. Acad. Sci. USA.

[86] Wälti M.A., Schmidt T., Murray D.T., Wang H., Hinshaw J.E., Clore G.M. (2017). Chaperonin GroEL accelerates protofibril formation and decorates fibrils of the Het-s prion protein. Proc. Natl. Acad. Sci. USA.

[87] Drulis-Kawa Z., Olszak T., Danis K., Majkowska-Skrobek G., Ackermann H.-W. (2013). A giant Pseudomonas phage from Poland. Arch. Virol..

[88] Abbasifar R., Griffiths M.W., Sabour P.M., Ackermann H.-W., Vandersteegen K., Lavigne R., Noben J.-P., Villa A.A., Abbasifar A., Nash J.H. (2014). Supersize me: Cronobacter sakazakii phage GAP32. Virology.

[89] Caubilla-Barron J., Hurrell E., Townsend S., Cheetham P., Loc-Carrillo C., Fayet O., Prère M.-F., Forsythe S. (2007). Genotypic and Phenotypic Analysis of Enterobacter sakazakii Strains from an Outbreak Resulting in Fatalities in a Neonatal Intensive Care Unit in France. J. Clin. Microbiol..

[90] Kabanova A.P., Shneider M.M., Korzhenkov A.A., Bugaeva E.N., Miroshnikov K.K., Zdorovenko E.L., Kulikov E.E., Toschakov S.V., Ignatov A.N., Knirel Y.A. (2019). Host Specificity of the Dickeya Bacteriophage PP35 Is Directed by a Tail Spike Interaction with Bacterial O-Antigen, Enabling the Infection of Alternative Non-pathogenic Bacterial Host. Front. Microbiol..

[91] Czajkowski R. (2019). May the Phage be with You? Prophage-Like Elements in the Genomes of Soft Rot Pectobacteriaceae: *Pectobacterium* spp. and *Dickeya* spp.. Front. Microbiol..

[92] Toth I.K., Van Der Wolf J.M., Saddler G.S., Lojkowska E., Helias V., Pirhonen M., Lahkin L.T., Elphinstone J.G. (2011). Dickeya species: An emerging problem for potato production in Europe. Plant Pathol..

[93] Pendleton J.N., Gorman S.P., Gilmore B.F. (2013). Clinical relevance of the ESKAPE pathogens. Expert Rev. Anti-Infect. Ther..

[94] Rosenthal V.D., Al-Abdely H.M., El-Kholy A.A., Alkhawaja S.A.A., Leblebicioglu H., Mehta Y., Rai V., Hung N.V., Kanj S.S., Salama M.F. (2016). International Nosocomial Infection Control Consortium report, data summary of 50 countries for 2010-2015: Device-associated module. Am. J. Infect. Control..

[95] Pires D.P., Costa A.R., Pinto G., Meneses L., Azeredo J. (2020). Current challenges and future opportunities of phage therapy. FEMS Microbiol. Rev..

[96] Lin D.M., Koskella B., Lin H.C. (2017). Phage therapy: An alternative to antibiotics in the age of multi-drug resistance. World J. Gastrointest. Pharmacol. Ther..

[97] Rohde C., Resch G., Pirnay J.P., Blasdel B.G., Debarbieux L., Gelman D., Górski A., Hazan R., Huys I., Kakabadze E. (2018). Expert Opinion on Three Phage Therapy Related Topics: Bacterial Phage Resistance, Phage Training and Prophages in Bacterial Production Strains. Viruses.

[98] São-José C. (2018). Engineering of Phage-Derived Lytic Enzymes: Improving Their Potential as Antimicrobials. Antibiotics.

[99] Fenton M., McAuliffe O., O’Mahony J., Coffey A. (2010). Recombinant bacteriophage lysins as antibacterials. Bioeng. Bugs.

[100] Breidenstein E.B., De La Fuente-Nunez C., Hancock R.E. (2011). Pseudomonas aeruginosa: All roads lead to resistance. Trends Microbiol..

[101] Lee K., Yoon S.S. (2017). Pseudomonas aeruginosa Biofilm, a Programmed Bacterial Life for Fitness. J. Microbiol. Biotechnol..

[102] Latz S., Krüttgen A., Häfner H., Buhl E.M., Ritter K., Horz H.-P. (2017). Differential Effect of Newly Isolated Phages Belonging to PB1-Like, phiKZ-Like and LUZ24-Like Viruses against Multi-Drug Resistant Pseudomonas aeruginosa under Varying Growth Conditions. Viruses.

[103] Can K., Aksu U., Yenen O.Ş. (2018). Investigation of PhiKZ phage therapy against Pseudomonas aeruginosa in mouse pneumonia model. Turk. J. Med Sci..

[104] Danis-Wlodarczyk K., Vandenheuvel D., Jang H.B., Briers Y., Olszak T., Arabski M., Wasik S., Drabik M., Higgins G., Tyrrell J. (2016). A proposed integrated approach for the preclinical evaluation of phage therapy in Pseudomonas infections. Sci. Rep..

[105] Polygach O.A., Dabizheva A.N., Voroshilova N.N. (2018). Effect of the Composition of Lytic Bacteriophages of P. aeruginosa Formation and Destruction of Bacterial Biofilms. Epidemiol. Vaccinal Prev..

[106] Paradis-Bleau C., Cloutier I., Lemieux L., Sanschagrin F., Laroche J., Auger M., Garnier A., Levesque R.C. (2007). Peptidoglycan lytic activity of the *Pseudomonas aeruginosa* phage phiKZ gp144 lytic transglycosylase. FEMS Microbiol. Lett..

[107] Briers Y., Walmagh M., Lavigne R. (2011). Use of bacteriophage endolysin EL188 and outer membrane permeabilizers against Pseudomonas aeruginosa. J. Appl. Microbiol..

[108] Walmagh M., Briers Y., Dos Santos S.B., Azeredo J., Lavigne R. (2012). Characterization of Modular Bacteriophage Endolysins from Myoviridae Phages OBP, 201φ2-1 and PVP-SE1. PLoS ONE.

